# MSQPSO-Optimized MSCC-CAE for Sensor Fault Detection and Localization in Small Modular Reactors

**DOI:** 10.3390/s26061916

**Published:** 2026-03-18

**Authors:** Weiwei Zhang, Xuesong Wan, Xueting Li, Zhengxi He, Maokang Luo

**Affiliations:** 1 School of Mathematics, Sichuan University, Chengdu 610065, China; 2021322010057@stu.scu.edu.cn (W.Z.); makaluo@scu.edu.cn (M.L.); 2 School of Aeronautics and Astronautics, Sichuan University, Chengdu 610065, China; 3 School of Big Data, Chengdu Technological University, Chengdu 611730, China; wxsong1@cdtu.edu.cn; 4 National Key Laboratory of Science and Technology on Reactor System Design Technology, Nuclear Power Institute of China, Chengdu 610213, China; hezhengxi@npic.ac.cn

**Keywords:** sensor fault detection and localization, small modular reactors, multi-scale correlation features, multi-scale cross-correlation-based convolutional autoencoder framework (MSCC-CAE), multi-strategy improved quantum particle swarm optimization (MSQPSO)

## Abstract

As small modular reactors (SMRs) evolve towards longer lifespans, autonomous operation, and high reliability, the accuracy and reliability of sensor data are crucial for ensuring the safe operation of nuclear power systems. To improve the accuracy of multi-source sensor fault detection and localization in small reactors, this paper proposes a multi-scale cross-correlation-based convolutional autoencoder (MSCC-CAE) framework. First, multiple sensor cross-correlation matrices are constructed across multiple time scales to explicitly characterize the dynamic coupling relationships between heterogeneous sensors. These multi-scale correlation features can effectively capture both short- and long-term dependencies among sensors. Then, a convolutional autoencoder is used to compress and reconstruct the correlation matrix, thereby learning low-dimensional discriminative representations for fault detection. To enhance the stability and generalization of the proposed framework, a multi-strategy improved quantum particle swarm optimization (MSQPSO) algorithm is proposed to adaptively optimize key network hyperparameters. Finally, the proposed method was validated using data from an SMR simulation model. Experimental results demonstrate that the proposed MSCC-CAE achieves a fault detection accuracy of 98.21%, outperforming CNN and conventional CAE models by 15.17 and 12.04 percentage points, respectively. The localization accuracy reaches 97.12%. These results verify the effectiveness and superiority of the proposed framework for intelligent sensor fault detection in the SMR system.

## 1. Introduction

A low-carbon, clean, and economically reliable energy supply is essential for high-quality development. As a key source of clean energy, nuclear power plays an important role in alleviating structural pressures during the transformation of the energy sector. In recent years, with the trend toward miniaturization and intelligentization of nuclear energy systems, SMRs have emerged as a major direction in international nuclear engineering research [1,2]. They have also become a strategic priority for many countries.

However, most SMRs are deployed on offshore platforms or in remote land-based areas, which creates a pressing demand for unmanned reactor operation [3] and imposes higher reliability requirements on operational monitoring and safety management systems. Sensors are crucial sources of information for reactor monitoring and safety assessment, providing real-time measurements of key parameters such as temperature, pressure, and flow rate. Sensor data accuracy and reliability directly affect the safety, reliability, and economic performance of nuclear power systems [4] and are vital for automatic control and online monitoring. However, sensors working long-term in complex high-temperature, high-pressure, and high-radiation environments can develop faults like drift and jamming [5], seriously harming data accuracy and reliability. Gradual faults are especially challenging because they evolve slowly with subtle early features, making them much harder to detect and locate [6,7]. Detecting these faults quickly and correctly is important to keep the system safe, reduce maintenance costs, and allow unmanned operation in SMRs.

In fault detection, redundancy analysis uses data from multiple sensors to check sensor status through information fusion [8]. Compared with single-sensor detection methods, these approaches more fully exploit inter-sensor correlations, offering greater reliability and robustness under complex operating conditions and noise interference, and have thus attracted extensive research interests. Based on their reliance on prior system models, redundancy analysis methods are generally categorized into model-driven and data-driven approaches [1].

Model-driven methods typically depend on high-precision mechanistic models [9]. However, for complex, multivariable, nonlinear, and strongly coupled systems such as nuclear power plants, model inaccuracies tend to accumulate as operating conditions change, making precise physical modeling difficult. Consequently, model-driven methods are often unsuitable for complex nuclear power scenarios [10].

In contrast, data-driven methods do not require physical modeling; instead, they employ operational data and machine learning algorithms to learn the underlying relationships among variables [11,12]. Previous studies have used traditional machine learning techniques, such as principal component analysis (PCA) [13,14], for sensor fault detection, achieving some improvements. However, these methods often rely on manually designed features [15], limiting their ability to construct generalizable, robust feature extractors for handling multi-source, strongly coupled sensor data from nuclear power systems.

With advances in deep learning, its multilayer, nonlinear modeling capability has opened new avenues for representing complex system states [16,17]. In multi-sensor scenarios, researchers have adopted multi-channel inputs or multivariate joint modeling to capture overall system behavior. For instance, Gallon et al. [18]. applied a multi-channel convolutional neural network (CNN) to jointly model spacecraft accelerometer and inertial measurement unit data for sensor fault detection. Chen et al. [19]. proposed a network combining multi-channel CNNs, squeeze-and-excitation (SE) attention, and an improved LSTM to extract spatial features from multi-sensor data and incorporate temporal modeling for chemical process diagnosis. Chen et al. [20]. introduced a three-layer autoencoder that takes raw multivariate time series as input for hierarchical feature learning, enabling high-precision detection of rolling bearing faults in aircraft engines under various conditions. Yet, most existing deep learning methods lack explicit modeling of sensor correlations and operate directly on raw multivariate time series. This limits their ability to capture subtle temporal changes in sensor relationships during gradual faults and restricts model interpretability and robustness.

To better capture correlation dependencies among multi-source sensors, some studies have begun integrating correlation structures into model inputs. Research has shown that cross-correlations between variables can represent system states more robustly than raw measurements [21,22,23]. For example, Liang et al. [23]. used a generative adversarial network to reconstruct cross-correlation matrices for anomaly detection in aerospace data. However, most correlation-based methods construct static representations on a single time scale or within fixed windows, failing to capture the dynamic, multi-scale evolution of sensor correlations, thereby limiting their effectiveness in gradual or incipient fault scenarios.

To effectively represent and analyze these dynamically evolving correlations, a structured data representation is required. The cross-correlation matrix, which quantifies pairwise sensor relationships, provides such a structure. However, it belongs to a grid-based data form, requiring machine learning techniques suitable for grid-based processing to extract its features [24]. Convolutional autoencoders (CAEs) have demonstrated strong performance in compressing and representing grid-based data (e.g., images, matrices) and have been widely used in industrial fault diagnosis [25,26,27,28]. By treating cross-correlation matrices as structured inputs and applying CAE for feature learning, dimensionality can be reduced while preserving key coupling information.

Moreover, hyperparameters of deep learning models are often set empirically, and performance is highly sensitive to these choices, affecting both results and tuning efficiency. Many researchers have adopted intelligent optimization methods—such as ant colony optimization [29], genetic algorithms [30], sparrow search algorithms [31], and particle swarm optimization (PSO) [32]—to tune deep model parameters [33]. Among these, the quantum particle swarm optimization (QPSO) algorithm offers strong global optimization and stability in high-dimensional, complex problems [34,35], but it still suffers from premature convergence and limited search diversity in practical engineering applications.

In summary, existing sensor fault diagnosis methods still have certain limitations in characterizing the coupling relationships of multi-source heterogeneous sensors and adaptively optimizing model hyperparameters. Therefore, this paper proposes a sensor fault detection and localization method that integrates multi-scale cross-correlation (MSCC) matrices, a convolutional autoencoder (CAE), and a multi-strategy improved QPSO (MSQPSO) for anomaly detection in multi-source nuclear power sensors. The main contributions are as follows:

1. An explicit multi-scale correlation modeling framework is proposed. Unlike traditional methods that independently process sensor signals or only implicitly capture inter-sensor dependencies, the MSCC-CAE framework constructs MSCC matrices at different time scales to explicitly represent the coupling relationships between heterogeneous sensors. By introducing structured prior information into the input representation, the ability to represent the state of complex systems is improved.
2. A multi-strategy improved MSQPSO algorithm is proposed to achieve adaptive configuration of network hyperparameters. Compared with the parameter tuning method that relies on manual experience, it improves the search capability and robustness of the hyperparameter optimization process, thereby enhancing the stability and generalization performance of the entire framework.
3. The effectiveness of the proposed methods is validated on the task of multi-source sensor fault detection and localization in an SMR. Experimental results show that the proposed approach outperforms several existing methods in terms of detection accuracy and reliability.

The remainder of this paper is organized as follows: Section 2 introduces the MSCC-CAE framework and methods based on MSQPSO; Section 3 describes the experimental setup; Section 4 reports the experimental results; and Section 5 concludes the paper.

## 2. Fault Detection Model Based on MSCC-CAE

### 2.1. Cross-Correlation Matrix

The correlations among variables can effectively characterize the system state, and normal perturbations in the data have minimal impact on these correlations [35]. The Cross-Correlation Matrix (CCM) is a statistical tool used to quantify the strength of relationships between different sensor signals. In this work, the CCM is calculated to capture the correlation features among multivariate signals, as shown in Equation (2). Let the time series of sensor i be $X_{i}=\{x_{i}^{0},x_{i}^{1}\ldots ,x_{i}^{t}\}(i=1,2,\ldots ,n)$. At time t, a segment of length T is extracted from $X_{i}$ as $\left\{x_{i}^{t-T+1}\ldots ,x_{i}^{t-1},x_{i}^{t}\right\}$. Then, the cross-correlation matrix $M_{t}^{T}$ at time t with time scale T is calculated as follows [36]:

(1) $$M_{t}^{T}=\left[\begin{array}{cccc} m_{11}^{T} & m_{12}^{T} & \ldots & m_{1n}^{T}\\ m_{21}^{T} & m_{22}^{T} & \ldots & m_{2n}^{T}\\ \vdots & \vdots & \ddots & \vdots \\ m_{n1}^{T} & m_{n2}^{T} & \ldots & m_{nn}^{T} \end{array}\right]$$

(2) $$m_{ij}^{T}=\frac{\sum_{\sigma =0}^{T}x_{i}^{t-\sigma }x_{j}^{t-\sigma }}{T}$$

where $m_{ij}^{T}$ represents the cross-correlation between sensors i and j over scale T.

**Figure 1:**
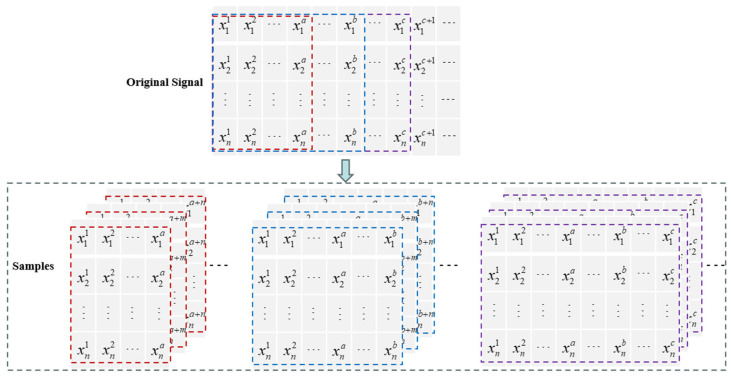
Illustration of Sample Generation Using the Sliding Window Method.

To capture dynamic features at different time scales, this paper employs a sliding window technique, as shown in Figure 1. Different window lengths correspond to dynamic information at different time scales, determined based on data characteristics and preliminary experimental observations. Three time scales were set: T = {10, 30, 60}. The multivariate time series is segmented into multi-scale data, and the CCM is calculated. The short time scale reflects short-term fluctuations in the data, while the long time scale reflects the overall trend and steady-state characteristics of the system, thus enhancing global representation capabilities. The fusion of multi-time scale information helps to improve the accuracy of system state identification and the timeliness of responses.

### 2.2. Convolutional Autoencoder

CAE [37] is an unsupervised neural network framework that extracts hierarchical features from input data using convolutional layers, while the decoder uses deconvolutional layers to reconstruct the features back into the input space [38]. Due to its characteristics such as local receptive fields, weight sharing, and translation invariance, CAE can effectively capture local spatial or temporal correlations, enabling unsupervised detection of sensor anomalies.

In this framework, the computed cross-correlation matrices are fed into the convolutional autoencoder. First, the input is progressively compressed through the convolutional encoding module. Assuming that the feature representation of the $\left(l-1\right)$-th layer is $E^{l-1}$, the feature representation of the l-th layer can be expressed as [39]:

(3) $$\left\{\begin{array}{c} E^{l}=\partial (K^{l}*E^{l-1}+b^{l})\\ E^{l}=CCM \end{array}\right.$$

where ∗ denotes the convolution operation, $k^{\left(l\right)}$ represents the convolution kernel of the l-th layer, $b^{\left(l\right)}$ denotes the bias term of the l-th layer, and $\partial \left(\cdot \right)$ is the activation function. During the encoding process, shallow convolutional layers extract local features from the CCM, while deeper convolutional layers further compress these features to obtain a low-dimensional representation of the CCM.

The convolutional decoding module adopts a structure symmetric to that of the encoder and progressively deconvolves the encoder output. The feature representation of the l-th layer in the decoder can be computed as follows:

(4) $$\left\{\begin{array}{c} D^{l}=\partial \left({\hat{K}}^{l}⊗D^{l-1}+{\hat{b}}^{l}\right)\\ D^{l}=E^{4}(l=0) \end{array}\right.$$

where ⊗ denotes the deconvolution (transposed convolution) operation, $k^{\left(l\right)}$ represents the convolution kernel of the l-th layer, and $b^{\left(l\right)}$ denotes the bias term of the l-th decoder layer.

The output of the final layer of the decoder is regarded as the reconstructed CCM, denoted as $CCM^{\prime }$.

### 2.3. MSCC-CAE Framework

**Figure 2:**
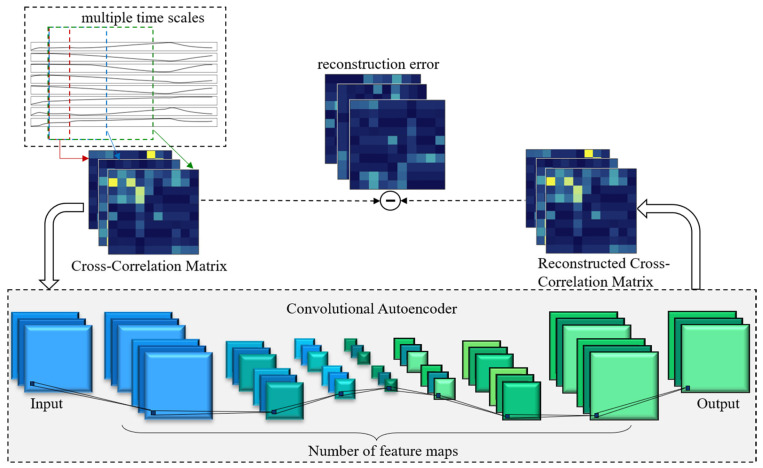
Architecture of the MSCC-CAE framework.

This paper proposes an MSCC-CAE framework for addressing the multi-source sensor-fault detection problem in SMRs. The architecture of the MSCC-CAE framework is shown in Figure 2.

Unlike methods that directly use raw multivariate time series as framework input, the proposed MSCC-CAE framework uses sensor-related structures as input, explicitly characterizing the changing relationships between multiple sensors.

MSCC-CAE processes multi-source sensor data using sliding windows at different time scales and constructs corresponding sensor cross-correlation matrices to explicitly represent the dynamic evolution of the sensor correlation structure at short- and long-term scales, thereby enhancing the framework’s ability to capture the coupling characteristics of complex systems. The constructed multi-scale correlation matrices are then used as input to a convolutional autoencoder for feature compression and reconstruction. Through the encoding-decoding process, the framework learns a discriminative representation in a low-dimensional latent space that reflects the normal correlation patterns of the system. In the fault detection stage, the system’s operating state is evaluated based on the reconstruction error of the correlation matrix. When a sensor fails and causes a significant deviation in the correlation structure, its reconstruction error increases, enabling the detection and localization of sensor faults.

### 2.4. MSQPSO-Based Hyperparameter Optimization

#### 2.4.1. MSQPSO Algorithm

In multi-source heterogeneous sensor fault diagnosis tasks, model optimization is crucial, especially when dealing with complex deep learning models such as convolutional autoencoders. Traditional hyperparameter optimization methods, such as grid search and random search, can find relatively good hyperparameter configurations, but they are often computationally intensive and inefficient [40]. Therefore, an algorithm that can automatically optimize hyperparameters is needed to improve the accuracy and robustness of the fault detection model.

The QPSO algorithm [41] theoretically offers advantages in global convergence compared to traditional PSO, but it still has certain limitations in practical applications:

(1) Insufficient global search capability

During the search process, QPSO updates particle positions primarily based on the individual historical best position and the global best position. This update mechanism tends to cause particles to distribute unevenly across the search space and to overly concentrate in the currently identified promising regions, thereby neglecting the exploration of other potentially optimal regions.

(2) Prone to premature convergence to local optima

As the number of generations increases, the particle swarm gradually converges toward the current best solution, leading to a rapid loss of population diversity. When multiple local optima exist in the search space, the algorithm is likely to become trapped in a local optimum and fail to escape, a problem that becomes particularly pronounced in high-dimensional, complex optimization tasks.

(3) Fixed strategy proportion

Conventional QPSO typically employs a fixed search strategy and lacks the ability to dynamically adjust the balance between exploration and exploitation as the swarm evolves during the optimization process. However, a static strategy lacks the flexibility required to adapt to different phases of the optimization process, which may lead to inadequate global exploration during the early stages and reduced convergence efficiency in the later stages.

To address these challenges, a multi-strategy version of the MSQPSO algorithm is presented. It combines several search strategies and uses evolutionary game theory to adjust their use based on the optimization stage, which helps balance global search and local search.

(1) Spiral Trajectory Exploration Strategy

To improve global search of QPSO, a spiral search strategy based on sparrow search is used, where particles move along spiral paths to cover a wider search space and avoid early convergence to local areas [42]. At the early stage of optimization, spiral movement lets particles search a wide space to find good regions, and as the process continues the movement becomes smaller so the algorithm keeps global search and also improves local search. The particle position update is defined as follows [43]:

(5) $$\left\{\begin{array}{c} x_{i}^{k+1}=zx_{r}^{k}\exp\left(\frac{-i}{c_{1}T}\right),c_{2}<ST\\ x_{i}^{k+1}=x_{r}^{k}+c_{3}zL_{1},c_{2}>ST \end{array}\right.$$

(6) $$z=\exp(r_{1}r_{2})\cdot \cos(2\pi r_{2})$$

where z denotes the spiral exploration factor, $x_{r}^{k}$ represents the particle position randomly selected at the k-th iteration, $c_{1}$ and $c_{2}$ are random numbers uniformly distributed in the interval [0,1], T is the maximum number of generations, ST denotes the warning constant, $c_{3}$ is a random variable following a standard normal distribution, $L_{1}$ represents the coefficient matrix, $r_{1}$ is the spiral shape constant, and $r_{2}$ is a random number uniformly distributed in the interval [−1, 1].

(2) Enhanced Local Search Strategy

An improved local search strategy based on QPSO is used, where particles search near the global best and past best positions to improve local search, explore nearby areas more accurately, reach good solutions faster, avoid getting stuck in small regions, and keep a better balance between global search and local refinement [44].

(7) $$x_{i}^{k+1}=gbest^{k}+c_{4}L_{2}(pbest_{r}^{k}-gbest^{k})\cdot L_{3}$$

where $c_{4}$ denotes the trajectory constant; $L_{2}$ is a position coefficient vector whose elements are independently drawn from a normal distribution with a mean of 0.5 and a standard deviation of 2; $pbest_{r}^{k}$ represents a historical best position randomly selected at the *k*-th iteration; and $L_{3}$ is a coefficient vector with the same dimensionality as the particle.

(3) Lévy Random Jump Strategy

The Lévy random walk strategy uses the Lévy distribution to set particle step sizes [35], which creates both small and large moves so particles can search nearby areas and also make long jumps, keep diversity in the search space, and reduce early convergence to local optima.

In high-dimensional or complex search spaces, the Lévy random walk lets particles search a wider range and reduces the limits of only local search. The implementation proceeds as follows [45]:

(8) $$s=\frac{u}{{\left|\nu \right|}^{1/\beta }}$$

(9) $$u∼N(0,\sigma_{u}^{2})$$

(10) $$\nu ∼N(0,\sigma_{\nu }^{2})$$

(11) $$\sigma_{u}=\frac{\Gamma (1+\beta )\sin(\pi \beta /2)}{2^{(\beta -1)/2}\Gamma [(1+\beta )/2]\beta }$$

(12) $$\sigma_{v}=1$$

where u and v are normally distributed variables, β is a randomly generated number within the interval [1.2, 1.7], and Γ is the standard gamma function.

Particle Position Update [46]:

(13) $$x_{i}^{k+1}=x_{i}^{k}+s(gbest^{k}-x_{i}^{k})$$

In many optimization algorithms, fixed particle strategies can reduce accuracy in complex problems, so a dynamic strategy based on evolutionary game theory is used, where entropy information lets particles change their strategies during the search and interact with others to keep a good balance between global and local search and improve overall performance.

In the proposed framework, each particle acts as a decision unit in a game with other particles and moves toward the best solution by updating its strategy, where fitness, past best positions, and entropy values are used to decide whether to keep or change the strategy, while boundary particles are updated first to reduce computation and avoid too many updates.

Assume that $S_{1},S_{2}$ and $S_{3}$ denote the three strategies introduced in Groups 1, 2, and 3, respectively, and the payoff matrix is $P=\left[P_{1},P_{2},P_{3}\right]$.

The average payoff for the entire group during the game is [47]:

(14) $$U=U_{1}+U_{2}+U_{3}=Z_{1}P_{1}+Z_{2}P_{2}+Z_{3}P_{3}$$

(15) $$Z_{1}+Z_{2}+Z_{3}=1$$

where *U* denotes the expected payoff of the entire population; $U_{1},U_{2}$ and $U_{3}$ represent the payoffs of Groups 1, 2, and 3, respectively; $Z_{1},Z_{2},Z_{3}$ denote the proportions of particles adopting each strategy; and *P*_1_, *P*_2_, *P*_3_ are the payoffs obtained by Groups 1, 2, and 3, respectively.

Throughout the optimization process, both population diversity and particle fitness play a crucial role in determining the performance of the algorithm. Therefore, the computation of population payoff should account not only for particle fitness but also for population diversity. To balance the contributions of diversity and fitness during optimization, two key indicators are adopted: diversity contribution and fitness contribution. The diversity contribution is calculated based on the IFE value, an important metric for quantifying population diversity. Through this mechanism, the particle swarm is encouraged to maintain sufficient exploratory capability across the entire search space rather than relying solely on the current best solution, thereby effectively avoiding premature convergence to local optima [48].

(16) $$P_{m}=E_{m}+F_{m}$$

(17) $$E_{m}=\frac{e_{m}-\mu_{e}}{\delta_{e}}$$

(18) $$F_{m}=\frac{f_{m}-\mu_{e}}{\delta_{e}}$$

(19) $$e_{m}=\frac{1}{N_{m}}\sum_{i=1}^{N_{m}}T_{mi}$$

(20) $$f_{m}=max(-F_{mi}),m=1,2,3$$

where $E_{m}$ and $F_{m}$ denote the normalized diversity payoff and fitness payoff of strategy group m, respectively; $e_{m}$ and $f_{m}$ represent the original diversity payoff and original fitness payoff of strategy group m; $N_{m}$ is the number of particles in strategy group m; $T_{mi}$ denotes the number of neighborhoods excluding the i-th particle; $F_{mi}$ is the fitness value of the i-th particle in strategy group m; $\mu_{e}$ and $\mu_{f}$ are the mean values of $e_{m}$ and $f_{m}$ in strategy group m, respectively; and $\delta_{e}$ and $\delta_{f}$ are the corresponding variances of $e_{m}$ and $f_{m}$ in strategy group m.

In the calculation of diversity gain, the focus is primarily on particles located outside the neighborhood range. These particles help maintain the population distribution during the optimization process and encourage the particle swarm to explore a larger solution space. Based on this calculation method, diversity gain is used to reflect the population’s exploration ability and prevent the search process from prematurely converging to a local region.

Therefore, the replicator dynamic equation is given as follows [48]:

(21) $$\left\{\begin{array}{c} \frac{dZ_{1}}{dt}=Z_{1}(U_{1}-U)=Z_{1}(-Z_{2}P_{2}-Z_{3}P_{3})\\ \frac{dZ_{2}}{dt}=Z_{2}(U_{2}-U)=Z_{2}(-Z_{1}P_{1}-Z_{3}P_{3})\\ \frac{dZ_{3}}{dt}=Z_{3}(U_{3}-U)=Z_{3}(-Z_{1}P_{1}-Z_{2}P_{2}) \end{array}\right.$$

By setting the value of the replication dynamic equation to zero, the above equation can be rewritten as [48]:

(22) $$\left\{\begin{array}{c} Z_{1}=-\frac{P_{3}}{P_{1}}Z_{3}\\ Z_{1}=-\frac{P_{2}}{P_{1}}Z_{2}\\ Z_{2}=-\frac{P_{3}}{P_{2}}Z_{3} \end{array}\right.$$

The proportion of each strategy in the next generation [49]:

(23) $$\left\{\begin{array}{c} Z_{1}=\frac{-P_{2}P_{3}}{P_{1}P_{2}-P_{1}P_{3}-P_{2}P_{3}}\\ Z_{2}=\frac{-P_{1}P_{3}}{P_{1}P_{2}-P_{1}P_{3}-P_{2}P_{3}}\\ Z_{3}=\frac{P_{1}P_{2}}{P_{1}P_{2}-P_{1}P_{3}-P_{2}P_{3}} \end{array}\right.$$

IFE and EGT are combined in MSQPSO to make the algorithm more robust, where IFE measures particle diversity and gives feedback to keep enough search range and prevent early convergence, and EGT adjusts the three search strategies based on fitness and diversity to balance global and local search and avoid too much focus on small areas, allowing stable operation and accurate local solutions.

#### 2.4.2. Hyperparameter Optimization

In the MSCC-CAE framework, the number of convolution layers sets the network depth and controls feature extraction, where more layers let the model learn complex features but increase computation and risk overfitting, so the layer number must balance learning ability and cost.

The time-scale parameter shows sensor data changes at different speeds, where short scales capture fast changes and long scales show overall trends, and optimizing it helps the model get useful information from all scales. Time window weights set the importance of each scale so the model reacts more steadily to different time features.

The learning rate and batch size are important hyperparameters that affect the model’s training process. The learning rate sets how much parameters change and affects speed and stability, with too high a rate causing oscillation and too low a rate slowing convergence, and the batch size sets how many samples update the model at once, with larger batches speeding training but possibly reducing generalization. This paper uses MSQPSO to jointly optimize the above hyperparameters to achieve a balance between training efficiency and model generalization performance. Based on this, the objective function is defined as a weighted combination of multiple optimization objectives:

(24) $$f(x)=\alpha \cdot {\mathrm{Loss}}_{\mathrm{train}}(x)+\beta \cdot {\mathrm{Loss}}_{\mathrm{validation}}(x)$$

Here, $x=\{L_{\mathrm{conv}},T,W_{\mathrm{window}},\eta ,B_{\mathrm{batch}}\}$ denotes the vector of hyperparameters to be optimized; $L_{\mathrm{conv}}$ represents the number of convolutional layers; T is the set of temporal scale parameters; $W_{\mathrm{window}}$ denotes the allocation of time window weights; η represents the learning rate; and $B_{\mathrm{batch}}$ denotes the batch size.

**Figure 3:**
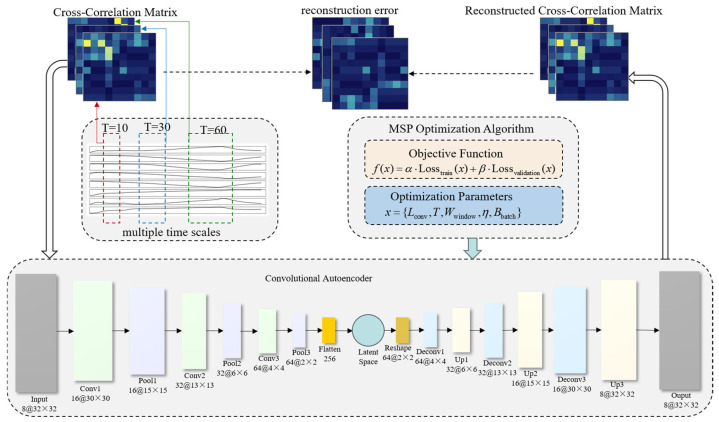
MSCC-CAE Sensor Fault Diagnosis Network Framework Optimized by MSQPSO.

**Figure 4:**
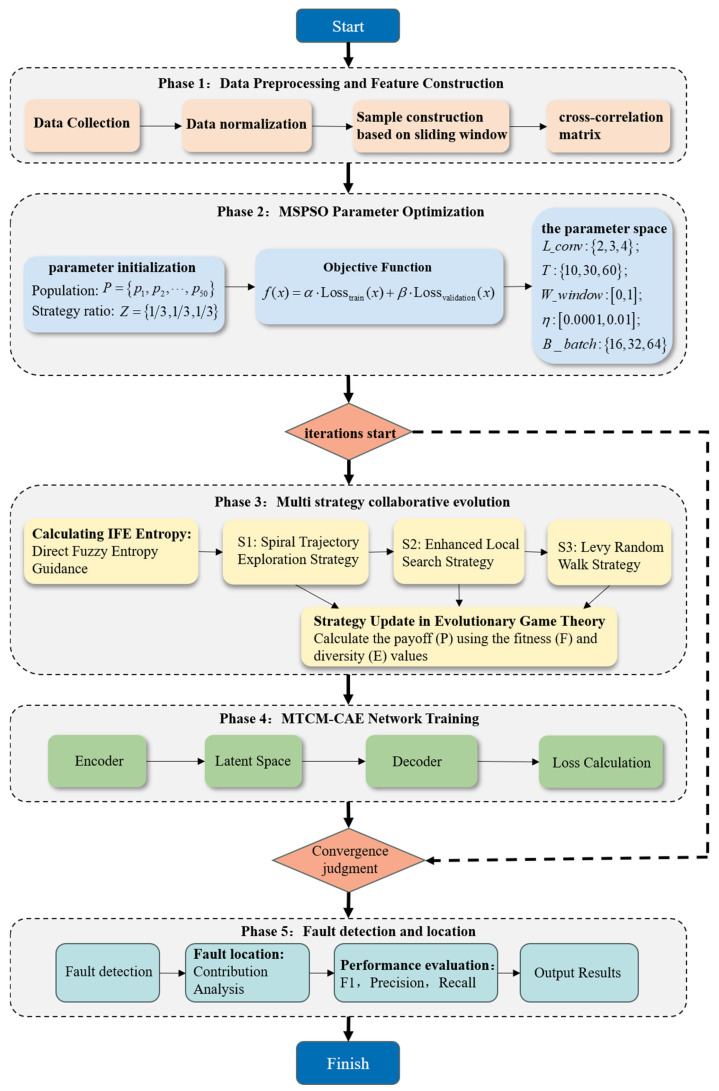
Workflow of the MSCC-CAE Sensor Fault Detection Algorithm Optimized by MSQPSO.

Based on the theoretical framework described above, this study develops a sensor fault detection and localization model for SMRs using the MSQPSO-optimized MSCC-CAE framework, as illustrated in Figure 3. Figure 4 shows the full workflow for sensor fault detection with two main parts: model training and fault diagnosis. The steps are:

Step 1: Prepare the dataset by splitting it into training, validation, and test sets, normalizing the data, and computing multi-scale cross-correlation matrices to capture sensor relationships at different times.

Step 2: Optimize MSCC-CAE hyperparameters using MSQPSO to improve feature extraction and model performance.

Step 3: Train the MSCC-CAE framework with the optimized hyperparameters to learn the correlations in normal sensor data.

Step 4: Set the fault detection threshold using the validation set by calculating error scores for normal samples and defining the threshold.

(25) $$theta=mean\left(S\_valid\right)+3*std\left(S\_valid\right)$$

This threshold distinguishes between normal and abnormal system states.

Step 5: CCM reconstruction and error computation. Input the CCM into the trained MSCC-CAE framework to obtain the reconstructed CCM. Compute the error score E_score between the original and reconstructed CCM, which quantitatively reflects the deviation of the system state from normal conditions at the current time.

Step 6: Fault detection. Compare the computed error score with the predefined threshold theta. If E_score > theta, the system is considered abnormal at the current time, triggering the fault localization procedure.

Step 7: Fault localization. When an anomaly is detected, calculate each sensor’s contribution to the total error. The contribution of each sensor is defined as the sum of the reconstruction errors of the CCM elements associated with that sensor. The sensor with the highest error contribution is identified as the faulty sensor, enabling precise fault source localization.

Step 8: Diagnostic result output. Output the fault diagnosis results, including whether a fault exists, the time of occurrence, and the location of the faulty sensor.

## 3. Experiments

### 3.1. Experimental Data

#### 3.1.1. SMR Description

This study takes the ACP100 small modular pressurized water reactor as the research object. The ACP100 is independently designed by the China National Nuclear Corporation and is suitable for multi-scenario energy supply applications. It is one of the first commercial demonstration SMRs worldwide to enter the construction design stage [40]. Unlike traditional pressurized water reactors, the ACP100 adopts an integrated main system layout, with the main primary loop structure integrated inside the reactor pressure vessel. Its main structures include the reactor core, steam generator, reactor pressure vessel, internal flow channels, and related in-core components, which reduces the complexity of the main loop and improves the compactness and safety of the reactor system.

The ACP100 sensor system primarily monitors key thermal–hydraulic parameters and state variables during reactor operation. The monitored variables typically include process parameters such as temperature, pressure, flow rate, and water level. These parameters are closely related to the operating status of the main system and related subsystems, characterizing the dynamic evolution of the reactor system.

For this type of sensor data, routine data integrity and reliability checks typically include cross-validation between physically redundant measurements, checking whether measured values exceed reasonable ranges, identifying abrupt signal changes or abnormally stable segments, and analyzing consistency with relevant process variables. Hardware redundancy is a common verification method that involves cross-comparing the same parameter using multiple redundant sensors to determine if anomalies exist in the measurement results. However, this method usually increases system costs, and its diagnostic capabilities remain limited in the presence of common-mode failures.

Because the various measurement signals in the ACP100 system originate from different but interrelated physical processes, the sensor data simultaneously exhibit significant temporal dynamic characteristics and varied coupling properties. Therefore, in addition to routine integrity checks, further utilizing multi-source sensor information fusion methods to model the correlation between signals can help improve the effectiveness of sensor fault detection and localization.

The experimental data used in this study were generated based on an ACP100 system simulation model built on the PCTRAN platform. Multiple sensors are deployed at key locations along the primary loop to monitor operational parameters, including pressure, temperature, and flow rate. Based on reactor operational characteristics and engineering experience, eight representative and non-redundant sensor parameters are selected, and their steady-state operating data are collected to form the experimental dataset, as listed in Table 1.

#### 3.1.2. Dataset Partitioning

The software simulation duration is 3000 s at a sampling frequency of 20 Hz, yielding 30,000 data samples. Among them, 70% of the data are used as the training set for model training, 15% as the validation set to determine the fault detection threshold, and the remaining 15% as the test set to evaluate the fault diagnosis performance of the proposed method.

#### 3.1.3. Fault Injection

Drift faults are among the most common types of incipient faults in sensors of nuclear power systems. Due to their small deviation amplitude and slow evolution in the early stage, drift faults are easily masked by normal operating fluctuations and measurement noise, making them particularly difficult to detect. Therefore, drift faults are selected as a representative fault scenario in this study to validate the proposed method’s capability for fault detection, faulty sensor localization, and fault-occurrence time identification under incipient fault conditions. The fault data are constructed based on simulation data of normal reactor operation. A mathematical model of the fault is established by combining typical sensor fault mechanisms in nuclear power instrumentation systems with expert experience. Faults are injected into the sensor signals by randomly selecting the fault occurrence time and fault parameters (such as drift slope) within a preset range. The mathematical expression for a drift fault is as follows [50]:

(26) $$y_{m}\left(t\right)=y_{g}\left(t\right)+k\left(t-t_{0}\right)$$

Here, k denotes the drift slope, and $t_{0}$ denotes the drift start time.

**Figure 5:**
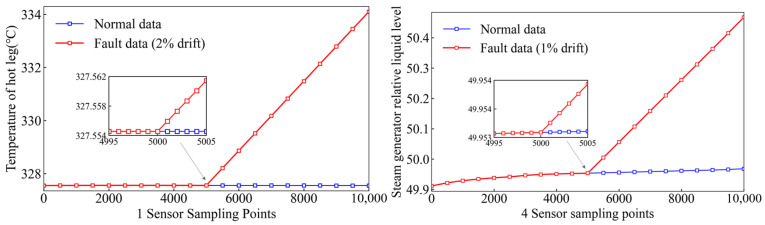
Comparison of faulty and normal sensor data.

The fault occurrence time $t_{0}$ is randomly selected within 20–80% of the total sampling interval to avoid boundary effects. The drift slope k is randomly sampled within ±(1–5%) of the nominal sensor value per time unit. Figure 5 shows the comparison between the normal and fault data for the two sensors.

### 3.2. Configuration of MSCC-CAE Framework

In this study, the parameter settings of the MSCC-CAE framework are critical to the experimental results. To validate the algorithm’s effectiveness, this paper configures the relevant parameters of MSCC-CAE and MSQPSO.

As shown in Table 2, a population size of 50 was set to ensure adequate coverage of the search space, and the maximum number of generations was set to 100. The inertia weight was linearly decreased from 0.9 to 0.4 to balance global exploration in the early stage and local fine-tuning in the later stage. The learning factors were selected to enhance the driving force of particles toward both individual and global optima.

As shown in Table 3, the search range for the number of convolutional layers was set to [2,5] to balance nonlinear feature extraction capability and model complexity. The temporal scale set was defined as $\left\{10,\;30,\;60\right\}$ to capture fault characteristics across different frequency domains. Weight allocations and learning rates were optimized in continuous spaces for fine-grained adjustment, while batch sizes were selected from commonly used discrete powers-of-two sets to optimize memory usage and training stability.

After optimization using the MSQPSO algorithm, the optimal hyperparameter configuration for the MSCC-CAE framework was obtained, as shown in Table 4. The optimization process converged after 100 generations, with a final fitness value of 0.0982, indicating that the model exhibited high performance on the validation set. This significantly improved the performance of the sensor fault diagnosis model and ensured its applicability across different datasets and tasks.

The optimization results indicate that: (1) increasing the number of convolutional layers from 3 to 4 enhanced the model’s feature extraction capability; (2) adopting a multi-temporal scale combination {10, 30, 60} allowed more comprehensive capture of fault characteristics compared to a single scale; (3) the temporal scale weight allocation of 0.25:0.45:0.30 indicates that the medium temporal scale (30) contributes most significantly to fault detection; (4) the learning rate was optimized to $3.27\times 10^{-4}$, balancing training speed and convergence stability; and (5) increasing the batch size to 64 improved training efficiency.

### 3.3. Fault Detection and Localization Strategies

This study focuses on the detection and localization of single-sensor faults. The proposed diagnostic strategy is developed based on the following criteria:

Let $m_{ij}^{T}$ denote the cross-correlation between sensor i and sensor j at temporal scale T, and let ${\hat{m}}_{ij}^{T}$ be the reconstructed value obtained from the MSCC-CAE framework. The error score *S* is then calculated using Equation (27):

(27) $$S=\sum_{i=1}^{n}s_{i}$$

(28) $$s_{i}=\sum_{j=1}^{n}\sum_{T=\{10,30,60\}}\left|m_{ij}^{T}-{\hat{m}}_{ij}^{T}\right|(i,j=1,2,\ldots ,n)$$

where $s_{i}$ represents the error contribution of the i-th sensor.

Compute the error scores for all samples in the validation set to form the sequence $S_{valid}$. The fault detection threshold is then defined as:

(29) $$threshold=mean(S_{valid})+3*std(S_{valid})$$

Here, mean(⋅) is used to calculate the mean of the sequence, and std(⋅) is used to calculate the standard deviation of the sequence.

During the fault detection process, if the error score of a sample at time t exceeds the threshold θ, the sample is considered abnormal; otherwise, it is considered normal. For abnormal points, the sensor with the largest error contribution is identified as the faulty sensor. Through this procedure, both the fault occurrence time and the location of the faulty sensor can be determined.

### 3.4. Model Evaluation Metrics

In the experiment, precision, recall, and F1 score were used to evaluate the model’s fault-detection performance. To evaluate the model’s spatial fault localization capability, Localization Accuracy (LA) is introduced. Suppose the dataset contains m samples, and let *a_i_* denote whether the localization of the i-th sample is correct. If the predicted label matches the true label, the localization is considered correct and $a_{i}=1$; otherwise, if the predicted label does not match the true label, the localization is considered incorrect and $a_{i}=0$.”

(30) $$a_{i}=\left\{\begin{array}{c} 1\cdot true\;label=predicted\;label\\ 0\cdot true\;label\;\neq \;predicted\;label \end{array}\right.$$

The localization accuracy of the model is then defined as:

(31) $$accuray=\frac{1}{m}\sum_{i=1}^{m}a_{i}$$

The value of localization accuracy ranges from 0 to 1, with higher values indicating better fault-localization performance.

Timeliness (TL) quantifies the number of samples correctly identified as faulty within the first 100 sampling points following the occurrence of a fault, corresponding to the true positives (TP) in this interval. The metric ranges from 0 to 100, with higher values indicating that the model can detect faults earlier.

## 4. Result Analysis

### 4.1. Comparative Experiments

To validate the proposed model’s performance, comparative experiments were conducted against six other models. Each model was tested eight times. In the k-th test, a linear drift fault with a maximum drift magnitude of 1% was introduced at the 2670-th data point of the sensor i. All evaluation metrics were averaged over the eight experiments to obtain the final performance assessment. To ensure the fairness of the comparative experiments, all methods used the exact same dataset partitioning scheme, and the detection threshold was determined on the validation set before evaluating the fault diagnosis performance on the test set.

Taking sensor 0 failure as an example, this study analyzes the effectiveness of the MSQPSO-optimized MSCC-CAE for fault detection and localization from three perspectives: the reconstructed error matrix from the CCM, the evolution of the error score, and the error contribution rate.

**Figure 6:**
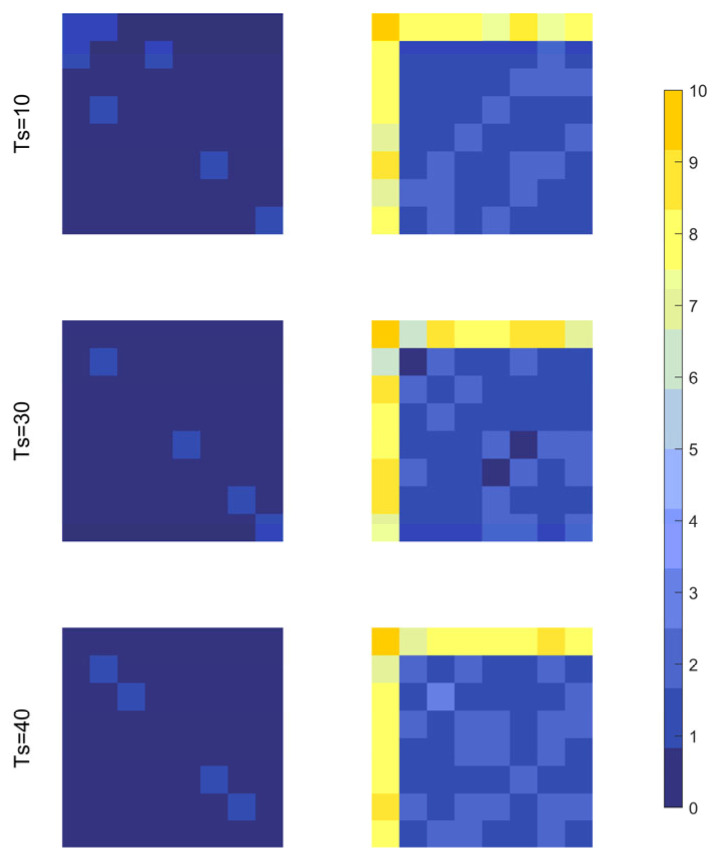
CCM reconstruction error matrices under normal and abnormal conditions.

Figure 6 shows the reconstruction error matrices of CCM at different time scales under normal conditions (2600th sampling point) and abnormal conditions (2800th sampling point). It can be seen that under normal conditions, the reconstruction errors of CCM at various scales are generally small, and no obvious abnormal clustering phenomenon is observed. In contrast, under abnormal conditions, the reconstruction error level increases significantly, and the larger errors are mainly concentrated in the 0th row and 0th column of the matrix, thus localizing the fault to sensor 0.

**Figure 7:**
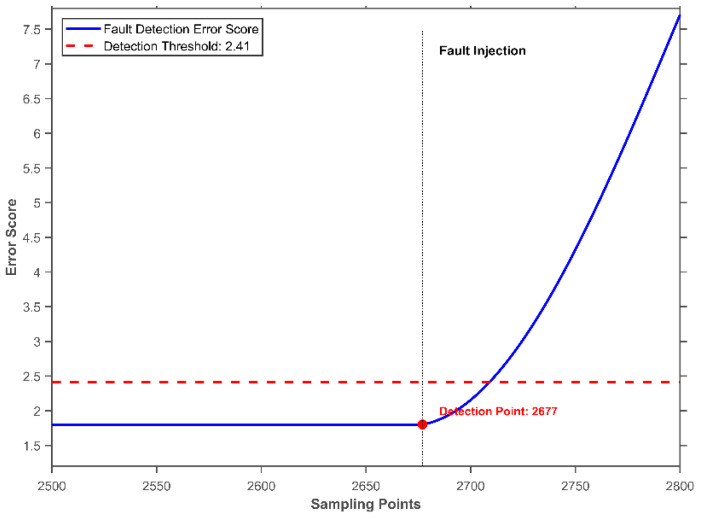
Error Score Curve under Fault in Sensor 0.

Figure 7 shows the error score curve for sampling points 2500–2800 in the test set. A fault was injected at sampling point 2677. After sensor 0 drifted, the error score exceeded the preset threshold starting from sampling point 2709, indicating that the sensor system had entered an abnormal state. Compared to the fault injection time, the detection result showed a delay of 32 sampling points, corresponding to approximately 0.35 s at a sampling frequency of 20 Hz.

**Figure 8:**
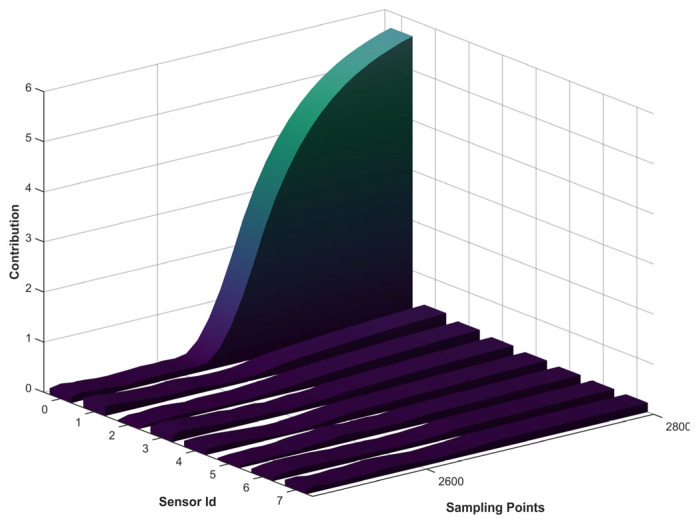
Contribution Ratios of All Variables under Fault in Sensor 0.

Figure 8 shows the contribution ratios of all sensors to the error when a fault occurs in sensor 0. According to the proposed localization strategy, once the sensor system is identified as abnormal, the sensor with the highest error-contribution ratio is deemed the fault source. As the drift magnitude increases, the error contribution ratios of all sensors increase, with sensor 0 exhibiting the highest contribution. Therefore, sensor 0 is identified as the faulty sensor.

To validate the superiority of the MSQPSO-optimized MSCC-CAE, Table 4 presents comparative experimental results against other commonly used fault detection models. As shown in Table 5, the MSQPSO-optimized MSCC-CAE achieves the best performance across all evaluation metrics, with significant advantages in detection accuracy (F1 = 0.9821), sensor localization accuracy (LA = 0.9712), and fault timing identification capability (TL = 94.625) over the other methods. The results show that the proposed method performs the detection and localization tasks more effectively for gradual sensor faults.

Table 5 presents the experimental results comparing the proposed model with other models. Among the compared models, PCA showed relatively low overall performance, with an F1 score of 0.7429, an LA of 0.7469, and a TL of 6.125, indicating its limited applicability in complex sensor fault scenarios. MSET showed improvement in localization accuracy (LA = 0.9296), but because it failed to effectively capture deep temporal features and account for sensor correlations, its F1 score and timeliness were only 0.7716 and 16.000, respectively, resulting in moderate overall performance.

In contrast, deep learning methods such as ANN, LSTM, and CNN further improved fault detection performance through nonlinear feature learning, achieving F1 scores of 0.8055, 0.8321, and 0.8304. The corresponding LA values were 0.9005, 0.9227, and 0.9358, and the TL values were 38.500, 50.732, and 56.584, respectively. Nevertheless, most of these methods are based on implicit representations of the original multivariate time series, and as a result, their ability to detect minor shifts in sensor correlations during slowly evolving drift faults is limited.

The GCN method, which explicitly leverages the underlying sensor topology, achieved enhanced performance, yielding an LA of 0.9469 and a TL of 58.420. However, its reliance on static or single-scale graph structures limited its ability to effectively characterize dynamic correlations, resulting in a recall rate of 0.8520, which did not reach the optimal level.

Among the above methods, MSQPSO-optimized MSCC-CAE achieved the best results across all evaluation metrics, with F1 scores, recall rates, localization accuracies, and timeliness reaching 0.9821, 0.9756, 0.9712, and 94.625, respectively. Compared to PCA, MSET, ANN, and LSTM, it achieved F1 scores of 23.92%, 27.05%, 17.66%, and 15.00%, respectively, and demonstrated significant advantages in multi-source sensor fault detection, faulty sensor localization, and fault occurrence time identification.

### 4.2. Ablation Experiment

#### 4.2.1. Impact of Multi-Scale Cross-Correlation Modeling

To further validate the effectiveness of the proposed multi-scale cross-correlation modeling strategy, a set of ablation experiments was designed while keeping the network architecture unchanged. Different correlation modeling approaches and temporal scale settings were compared, and the experimental results are presented in Table 6.

As shown in Table 5, the baseline CAE without explicit correlation modeling achieves an F1 score of 0.8617, LA of 0.9266, and TL of 59.213. Introducing single-scale cross-correlation matrices (CCM-CAE) with different temporal scales improves fault detection performance. However, performance varies across scales, indicating that fixed-scale approaches may not fully capture all relevant inter-sensor dynamics.

Compared to other methods, the MSCC-CAE model performed best, achieving an F1 score of 0.9553, recall of 0.9362, and precision of 0.9471, with a task completion rate of 81.732%. Relative to the baseline CAE model, the MSCC-CAE achieved improvements of 10.86%, 2.23%, and 38.08% in F1 score, LA, and TL, respectively. These results show that using multi-scale correlation helps the model capture sensor relationships over time, making it more sensitive to gradual faults and improving the accuracy and reliability of multi-source sensor fault diagnosis.

#### 4.2.2. Performance Validation of the MSQPSO Algorithm

To study how each improvement affects performance, ablation tests were done with the MTCM-CAE structure fixed, comparing versions optimized by QPSO, SQPSO1 with spiral search, SQPSO2 with better local search, SQPSO3 with Lévy jumps, and MSQPSO. Each variant was independently executed 30 times, and convergence behavior as well as stability were evaluated at the 20th, 50th, and 100th generations through statistical analysis.

(1) Convergence Analysis of the MSQPSO Algorithm

**Figure 9:**
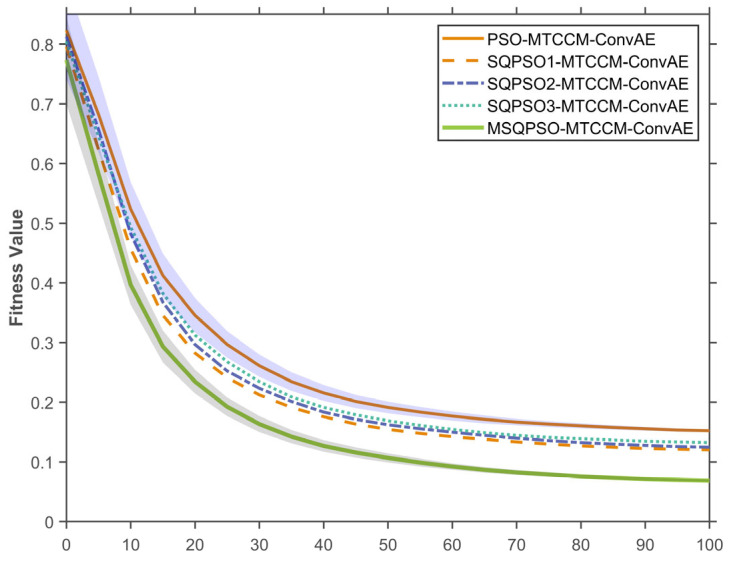
Fitness Convergence Curves.

Figure 9 illustrates the fitness convergence curves for the different algorithms. In this figure, the solid lines represent the mean fitness values across 30 independent runs, while the shaded areas surrounding the lines denote the standard deviation. This shaded region provides a visual measure of the algorithm’s stability and consistency during the optimization process. Overall, all algorithms showed high fitness values in the early stages of iteration, then gradually decreased as generations increased, and stabilized in the later stages.

QPSO-optimized MTCM-CAE (orange solid line) showed a rapid decrease in fitness in the initial stage, but the rate of decrease slowed significantly as the generations progressed, eventually stabilizing around 0.2. This result indicates that the traditional PSO-MTCM-CAE can obtain a relatively good solution within a limited number of generations, but it is easily trapped in local optima in the later stages of optimization.

SQPSO1-optimized MTCM-CAE (yellow dashed line, which introduces the spiral trajectory exploration strategy) showed stronger global search capabilities in the early generations, with a faster decrease in fitness than QPSO-optimized MTCM-CAE. As the generations continued, the convergence of this algorithm became smoother, and the final fitness decreased to below 0.2, indicating that spiral trajectory exploration enhanced global search capabilities to some extent, but its overall convergence efficiency remained lower than that of some improved strategies.

SQPSO2-optimized MTCM-CAE (blue dashed line, enhanced local search strategy) showed a similar trend to PSO-MTCM-CAE in the initial iteration stage, but its fitness decrease was smoother and lasted longer, reflecting better local optimization. In the later stages of iteration, the algorithm’s fitness stabilized around 0.1, indicating that the enhanced local search strategy further refines the local optimal solution.

SQPSO3-optimized MTCM-CAE (green dashed line, Levy random jump strategy) performs best, with fitness decreasing faster than for all other strategies. In the early stage, the Levy random-jump strategy effectively avoids local optima by introducing random jumps, leading to a rapid decline in fitness. In later generations, the fitness quickly stabilizes at a low value (close to 0.1), indicating superior performance in both global search and convergence speed.

Finally, MSQPSO-optimized MTCM-CAE (purple solid line) exhibits the fastest convergence, reflecting its strong combined ability for local refinement and global exploration.

(2) Stability Analysis of the MSQPSO Algorithm

**Figure 10:**
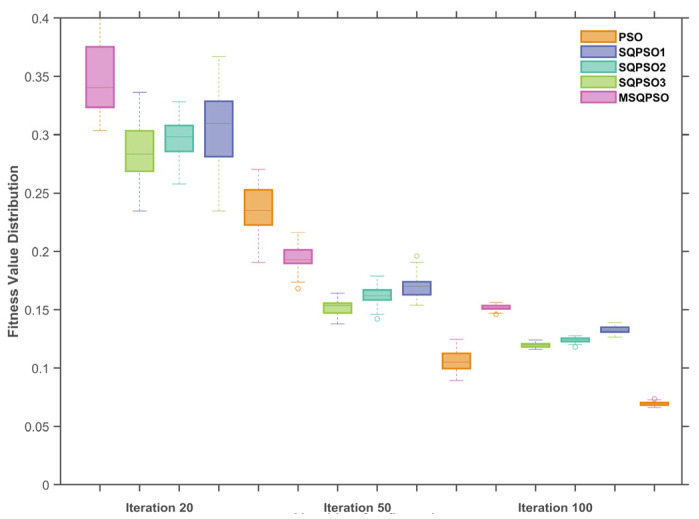
Boxplots at the 20th, 50th, and 100th generations.

As shown in Figure 10, the boxplots illustrate the fitness distributions of QPSO-optimized MTCM-CAE and different strategies (SQPSO1-optimized MTCM-CAE, SQPSO2-optimized MTCM-CAE, SQPSO3-optimized MTCM-CAE, and MSQPSO-optimized MTCM-CAE) at the 20th, 50th, and 100th generations. The boxes represent the interquartile range (IQR), the central line indicates the median, and the dashed whiskers extend to represent the data distribution range (typically 1.5*IQR). These whiskers effectively show the spread of the results and the presence of potential outliers. Specifically, the isolated circles plotted beyond the ends of the whiskers represent these outliers, highlighting individual data points that fall outside the expected distribution range.

At the 20th generation, all algorithms exhibit relatively high fitness values. QPSO-optimized MTCM-CAE shows the widest fitness distribution, indicating strong initial exploration capability but a lack of fine-tuning in subsequent generations. In contrast, SQPSO1-optimized MTCM-CAE and SQPSO2-optimized MTCM-CAE exhibit more concentrated fitness distributions at the 20th generation, suggesting they converge more precisely to superior solutions early in the process.

At the 50th generation, the fitness distributions of all algorithms gradually become more concentrated. MSQPSO-optimized MTCM-CAE shows the tightest distribution, indicating a smooth and efficient convergence. By comparison, SQPSO1-optimized MTCM-CAE and SQPSO2-optimized MTCM-CAE exhibit wider distributions, reflecting larger fluctuations during convergence.

At the 100th generation, the fitness distributions for all algorithms are even more concentrated. MSQPSO-optimized MTCM-CAE demonstrates the best convergence performance, achieving the lowest and most concentrated fitness values, highlighting its strong global search capability and high precision. Other strategies, such as SQPSO3-optimized MTCM-CAE, although improved relative to QPSO-optimized MTCM-CAE, still lag behind in terms of fitness concentration and precision.

Through the analysis of fitness convergence curves and box plots of QPSO-optimized MTCM-CAE and its improved strategies (SQPSO1-optimized MTCM-CAE, SQPSO2-optimized MTCM-CAE, and SQPSO3-optimized MTCM-CAE), it can be seen that MSQPSO-optimized MTCM-CAE exhibits lower fitness levels at the 20th, 50th, and 100th generations. Its convergence process decreases rapidly and stabilizes in the later stages within a range close to 0.1. Compared with other strategies, MSQPSO-optimized MTCM-CAE can reduce the fitness value more quickly in the early generations while maintaining a smaller fluctuation range in the later generations, indicating that this algorithm achieves a better balance between search efficiency and convergence stability. This characteristic makes it easier to obtain stable solutions in complex optimization spaces, thus providing reliable parameter configurations for subsequent fault diagnosis tasks.

## 5. Conclusions

This paper proposes an MSCC-CAE framework to address the problems of fault detection, localization, and fault occurrence time identification in SMRs. Unlike models that rely on reconstructing the original signal or implicitly learning sensor relationships through a network, the proposed method explicitly characterizes the dynamic coupling relationships between heterogeneous sensors by constructing a multi-scale cross-correlation matrix, thereby achieving a joint representation of short-term and long-term dependent features. Subsequently, CAE is used to compress and reconstruct the correlation matrix to learn discriminative low-dimensional features, which enables accurate detection and localization of sensor faults. Furthermore, to improve model stability, generalization, and hyperparameter optimization efficiency, an MSQPSO algorithm is proposed to adaptively optimize key hyperparameters. Experimental results based on SMR simulation data show that the proposed method achieves a fault detection accuracy of 98.21% and a localization accuracy of 97.12%, demonstrating higher detection accuracy and better reliability compared to traditional methods.

However, this research still has certain limitations. First, current research mainly focuses on drift faults and has not yet systematically considered other types of sensor faults such as bias faults, abrupt change faults, and jamming faults. Future research will focus on the detection and classification of multi-type and compound faults.

## Figures and Tables

**Table 1:** Key Sensor Parameters of SMR.

Number	Sensor Parameters	Units	Range
0	Flow of hot leg	kg/s	4903.73–4949.15
1	Temperature of hot leg	°C	325.42–328.76
2	Temperature of cold leg	°C	280.56–283.83
3	Steam generator pressure	MPa	6.32–6.71
4	Steam generator relative liquid level	1	45.15–55.17
5	Steam flow of steam generator	kg/s	492.9–558.6
6	Relative level of pressurizer	1	0.52–0.57
7	Pressure of pressurizer	MPa	15.07–15.48

**Table 2:** MSQPSO Parameter Settings.

Parameters	Settings
Population Size	50
Maximum Generations	100
Inertia Population	0.9->0.4 (Linear decrease)
Learning Factor	$c_{1}=1.5,\;c_{2}=2$

**Table 3:** Search Space of Hyperparameters to be Optimized.

Parameters	Search Space	Parameter Type
Number of Convolution layers	[2, 3, 4, 5]	Discrete
Weight distribution	[0.1, 0.9]	Continuous
Learning rate	$[10^{-4}$$,\;10^{-2}$]	Continuous
Batch size	{16, 32, 64, 128}	Discrete
Time-scale set	{10, 30, 60}	Discrete

**Table 4:** Optimal Parameter Configuration of MSCC-CAE Framework after MSQPSO.

Parameters	Before Optimization	After Optimization
Number of Convolution layers	3	4
Time-scale set	{30}	{10, 30, 60}
Weight distribution	0.5	0.25,0.45,0.30
Learning rate	1× 10^−3^	3.27 × 10^−4^
Batch size	32	64
Batch size	0.2156	0.0982
Number of Convolution layers	3	4
Time-scale set	{30}	{10, 30, 60}

**Table 5:** Fault Detection Results of Different Models.

Model	P	R	F1	LA	TL
PCA	0.7257	0.7611	0.7429	0.7469	6.125
MSET	0.7651	0.7784	0.7716	0.9296	16.000
ANN	0.8164	0.7950	0.8055	0.9005	38.500
LSTM	0.8373	0.8271	0.8321	0.9227	50.732
CNN	0.8453	0.8161	0.8304	0.9358	56.584
GCN	0.8711	0.8520	0.8614	0.9469	58.420
MSQPSO-MSCC-CAE	1.0000	0.9756	0.9821	0.9712	94.625

**Table 6:** Results of Multi-Scale Cross-Correlation Modeling with Different Scales.

Model	P	R	F1	LA	TL
CAE	0.8573	0.8663	0.8617	0.9266	59.213
CCM-CAE(ts = 10)	0.91189	0.8937	0.9027	0.9412	73.324
CCM-CAE(ts = 30)	0.9325	0.9220	0.99272	0.9368	71.250
CCM-CAE(ts = 60)	0.8958	0.8763	0.8859	0.9221	68.585
MSCC-CAE	0.9752	0.9362	0.9553	0.9471	81.732

## Data Availability

Data are contained within the article. All data in this paper are 564 generated by simulation and the details have been presented in Section 3.
